# Temperature-Optimized Liquid-Phase Iodide Ligand Exchange Enables Low-Trap Solution-Processed PbS Quantum Dot Photodetection at 940 nm

**DOI:** 10.3390/nano16060380

**Published:** 2026-03-22

**Authors:** Kapil Patidar, Her-Yih Shieh, Hsueh-Shih Chen

**Affiliations:** 1 Department of Materials Science & Engineering, National Tsing Hua University, Hsinchu 30013, Taiwan; 2 College of Semiconductor Research, National Tsing Hua University, Hsinchu 30013, Taiwan; 3 Department of Chemical Engineering & Materials Science, College of Engineering, Yuan Ze University, Taoyuan 32003, Taiwan

**Keywords:** PbS, QDs, ligand exchange, sensing, low-power, photodetector

## Abstract

PbS quantum dots (QDs) synthesized with oleic acid (OA) ligands suffer from poor charge transport in solid films, necessitating ligand exchange to shorter halide ligands for optoelectronic applications. This study investigates how ligand-exchange temperature governs OA-to-iodide substitution in PbS QDs. At 40 °C, the QD surface shows maximized halide passivation (I/Pb = 0.60) and minimized oxygen-related species (O/Pb = 0.23), suggesting reduced oxygen-associated defect formation and enabling n-type band alignment and reduced trap-mediated losses. PbS QD photodetectors fabricated from the 40 °C-treated QDs have 52% external quantum efficiency (EQE) at 940 nm (vs. 39% at 25 °C), with a responsivity of 0.394 A/W and an estimated detectivity of 2.1 × 10^13^ Jones. Temperature optimization of ligand-exchange provides a straightforward lever to improve device performance and reproducibility.

## 1. Introduction

Quantum dot (QD) optoelectronic materials and devices have attracted increasing attention in the past decades [1,2,3,4,5,6]. In parallel, near-infrared (NIR) photodetectors (PDs) are enabling components for emerging applications such as biometrics, time-of-flight (ToF) sensing, night vision, and wearable health monitoring, where high sensitivity around the 940 nm window is particularly important [7,8]. While conventional semiconductor PDs can deliver high performance, practical deployment is often limited by material and manufacturing constraints. For example, silicon devices suffer reduced responsivity near the band-edge, whereas epitaxial InGaAs platforms remain costly and less scalable [9,10,11]. Solution-processed colloidal quantum dots (QDs) provide an attractive alternative because their bandgap and absorption can be engineered by size and composition, and QD solids can be deposited over large areas using low-temperature processes [12,13,14,15]. Among the available QD materials, PbS QDs are especially promising for NIR photodetection because they offer strong absorption and tunable spectral response spanning the NIR to SWIR [16,17,18,19,20].

A central challenge for PbS QD PDs is that device performance is highly sensitive to surface chemistry and defect passivation [21,22,23,24]. As-synthesized PbS QDs typically carry long-chain oleic acid (OA) ligands that ensure colloidal stability in nonpolar solvents [25,26,27], yet these insulating ligands impede interdot electronic coupling and limit carrier transport in QD films [28,29]. Consequently, ligand exchange that replaces OA with shorter ligands or inorganic ions is essential to form compact, electronically coupled, and well-passivated PbS QD solids [30,31,32]. However, ligand exchange is governed by a competitive adsorption–desorption equilibrium and exchange kinetics, meaning that processing conditions can strongly influence iodide incorporation, oxygen-related surface species, and the resulting electronic energetics and trap density [33,34,35]. Solid-state layer-by-layer (LbL) exchange is the most widely adopted approach, yet repeated solvent exposure and film contraction may cause mechanical stress and non-uniform passivation in thick absorber films [26,31,33]. Solution-phase ligand exchange (SPLE) offers a practical alternative, enabling one-step deposition of compact films from pre-exchanged inks with reduced process complexity [23,26,30,35]. While ligand precursor concentration, solvent polarity, and exchange duration have been explored as optimization parameters, the QD solution concentration during SPLE has also been shown to strongly influence the QD surface chemistry. For example, optimizing ink preparation conditions can yield more complete OA removal and enhanced halide passivation, directly improving the performance of PbS QD solar cells [36]. However, the role of reaction temperature in governing exchange kinetics and surface passivation quality has received comparatively limited systematic attention.

In this work, we investigate and optimize the reaction temperature of a liquid-phase OA-to-iodide ligand exchange to reproducibly prepare PbS-I inks and high-quality films. We directly correlate surface composition, band energetics, and trap density with photodetection performance, demonstrating that an optimized exchange condition yields iodide-rich, low-oxygen surfaces with reduced trapping and improved NIR photodetection.

## 2. Experimental Section

### 2.1. Materials

Lead oxide (PbO, 99.9%) was purchased from ALFA Chemistry (Ronkonkoma, NY, USA). N,N-diphenylthiourea (98%), Lead iodide (PbI_2_), butylamine (BTA), acetonitrile (ACN), zinc acetate dihydrate (CH_3_COOZn‧2H_2_O), ammonium acetate (NH_4_Ac), dimethylformamide (DMF), and potassium hydroxide (KOH), were purchased from Sigma-Aldrich (St. Louis, MO, USA). 1,2-Ethanedithiol (EDT) was purchased from Tokyo Chemical Industry (Tokyo, Japan). Oleic acid (OA, 90%) was sourced from Li Yung Far (Taiwan). Diglyme (98%) was acquired from Vertex (Taiwan). N-octane was obtained from Echo Chemical (Taiwan). Dodecane (95%), Toluene (>99%), and methanol (>99%) were procured from Grand Chemical Co. Ltd. (Taiwan). Acetone (>99%) was obtained from Union Chemical (Taiwan).

### 2.2. Synthesis of PbS QDs

Synthesis of PbS QDs may be found in previous studies [33,37]. PbS QD synthesis was conducted under inert atmosphere conditions using a Schlenk system to minimize oxidation during nanocrystal formation. Briefly, 2 mmol of N,N-diphenylthiourea was dissolved in 10 mL of diglyme at room temperature, resulting in the formation of the sulfur precursor. Pb(OA)_2_ was prepared from lead oxide (PbO) reacting with oleic acid (OA), followed by adding 180 mL of dodecane. The sulfur precursor was injected into the Pb precursor at 95 °C in a three-neck flask, and the reaction proceeded for 10 min. The cooled solution was washed with acetone, followed by redispersing in toluene. The subsequent ligand exchange was performed in air.

### 2.3. Synthesis of ZnO NPs

Initially, Zinc acetate dihydrate (Zn(OAc)_2_·2H_2_O, 13 mmol) was dissolved in methanol at 60 °C and stirred for 10 min to serve as the zinc precursor [38,39,40,41]. Subsequently, Potassium hydroxide (KOH, 26 mmol) was dissolved in methanol, and this solution was added to the zinc precursor. After injection, the reaction temperature was raised to 65 °C and maintained for 90 min to allow for the coarsening of the ZnO nanoparticles. The resulting ZnO nanoparticles were purified by dissolving them in methanol. The purified ZnO nanoparticles were then dissolved in isopropyl alcohol (IPA) to create a solution with a concentration of 30 mg/mL. Subsequently, a small quantity of ethanolamine (EA, 0.1 vol%) was added to the ZnO nanoparticle solution, resulting in the formation of a highly transparent solution.

### 2.4. Preparation of the n-Type PbS-I Ink

PbS-I ink was prepared by solution-phase ligand exchange following a modified version of previously reported methods. Initially, 1 mmol of PbI_2_, and 0.4 mmol of ammonium acetate (NH_4_Ac) were dissolved in 5 mL of DMF as the iodide precursor. Subsequently, the PbS-OA QD solution (10 mg/mL in 5 mL n-octane) and the iodide precursor were vigorously mixed and heated at different temperatures (25–60 °C) in a water bath for 5 min. The ligand-exchanged quantum dot solution was separated and purified by centrifugation (5000 rpm for 5 min). Finally, the QDs were re-dissolved in a mixture of BTA and DMF for the device.

### 2.5. Device Fabrication

Prepatterned ITO glass was cleaned using deionized water, ammonium hydroxide, acetone, and isopropanol in an ultrasonic bath for 45 min, followed by UV-ozone treatment for 20 min. ZnO nanoparticles were spin-coated on top of the ITO glass in air at 3000 rpm for 42 s, followed by heat treatment at 150 °C for 10 min. PbS-I QDs after ligand exchange were dispersed in a solution containing butylamine (BTA) and dimethylformamide (DMF) for the deposition of the PbS active layer in a volume ratio of 4:1, deposited by spin coating at 3000 rpm for 27 s, followed by 60 °C heat treatment for 15 min. Then, two layers of PbS QDs (octane, 50 mg/mL) with 1,2-ethanedithiol (acetonitrile, 0.02 vol%) solid-phase ligand exchange treatment were deposited by spin coating at 3000 rpm for 42 s, followed by 60 °C heat treatment for 15 min. The top electrodes, which consisted of molybdenum trioxide (MoO_3_) and aluminum (Al) with thicknesses of 30 nm and 100 nm, respectively, were fabricated using thermal evaporation. The effective area of the device was 0.04 cm^2^. SCLC measurements were performed using the simple ITO/PbS-I/Al structure. The trap-filled-limit voltage (V_TFL_) was determined from the transition to the TFL regime in the log-log J-V curves. Trap-state density was estimated using $N_{t}=\frac{2\varepsilon \varepsilon_{0}V_{TFL}}{qL^{2}}$, where *L* is the PbS-I film thickness (~200 nm) and ε is the effective dielectric constant (~18).

### 2.6. Measurements and Characterizations

Fourier transform infrared spectroscopy (FT-IR) spectra were acquired using a Horiba F-730 spectrometer (Horiba, Kyoto, Japan) with a 2 cm^−1^ resolution. UV-Vis absorption was measured with a HITACHI U-3900 spectrometer (Hitachi, Tokyo, Japan). The ultraviolet photoelectron spectroscopy (UPS) was measured using the ULVAC-PHI PHI 5000 Versaprobe II instrument located at the Department of Materials Science and Engineering, National Tsing Hua University (Hsinchu, Taiwan) with He Iα radiation at an energy of 21.22 eV. XPS analysis was performed using an ULVAC-PHI PHI 5000 Versaprobe II with an Al Kα source located at the Department of Materials Science and Engineering, National Tsing Hua University (Hsinchu, Taiwan), calibrated to the C 1s peak (284.8 eV), and element ratios were calculated using following equation.

(1) $${XPS}_{elemental\;ratio}=\frac{I_{element}/S_{element}}{I_{sulfur}/S_{sulfur}}=\frac{{XPS}_{element}\%}{{XPS}_{sulfur}\%}$$

*I_element_* is the integrated area of the element, *I_sulfur_* is the integrated area of sulfur, *S_element_* is the relative sensitivity factor of the element, *S_sulfur_* is the relative sensitivity factor of sulfur. Current-voltage characteristics of the PD devices were recorded using a Keithley 2450 source meter (sweep mode) (Tektronix, Taiwan). The External Quantum Efficiency (EQE) data were recorded using certified equipment (Enli Technology, Kaohsiung, Taiwan) with monochromatic light from a xenon arc lamp.

## 3. Results and Discussion

### 3.1. Ligand Exchange and Device Fabrication

**Figure 1:**
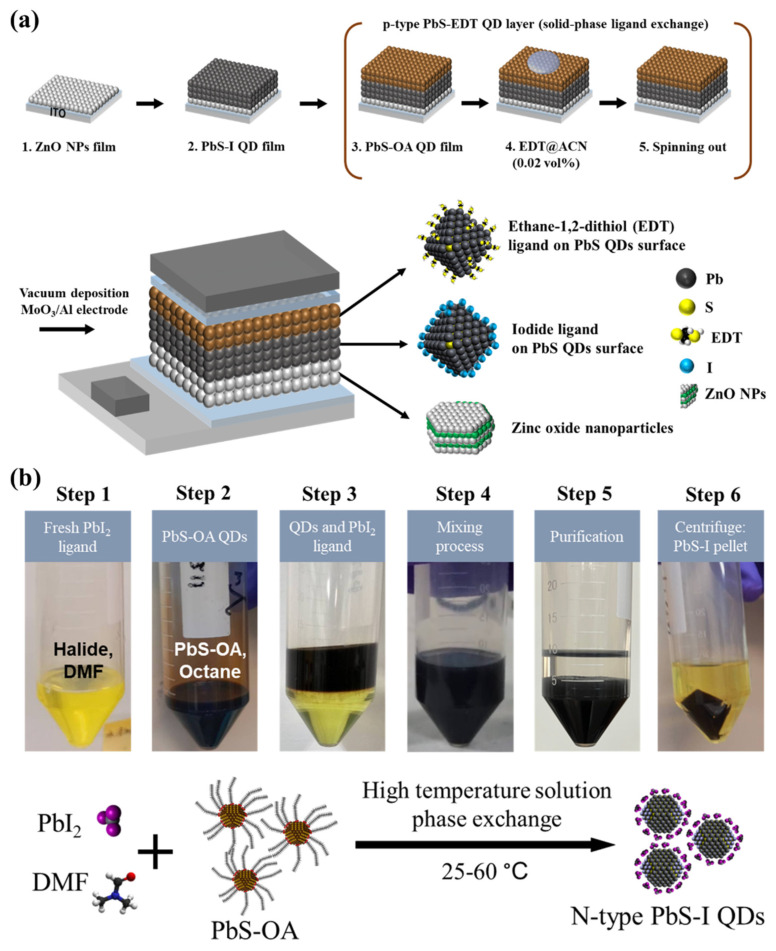
Ligand exchange and device fabrication of PbS QDs. (**a**) PbS QD PD device stack process with p-type PbS-EDT QD layer fabrication. (**b**) SPLE temperature processes for n-type PbS-I QDs.

The PD adopts a normal architecture of ITO/ZnO/PbS-I/PbS-EDT/MoO_3_/Al, as shown in the inset of Figure 1a. ZnO serves as the electron-transport layer, while the PbS-I/PbS-EDT bilayer forms an asymmetric n-p junction for efficient carrier separation and selective extraction. The PbS-I layer operates as the n-type, halide-passivated IR-absorbing and transport layer, where iodide ligands replace insulating OA to reduce surface traps and improve interdot charge transport. Although PbS-I films are often fabricated by solid-state LbL ligand exchange (spin-coat followed by iodide treatment and rinse, repeated cycles), the repeated solvent exposure and ligand-induced film contraction can accumulate mechanical stress and promote non-uniform exchange, which becomes increasingly detrimental when constructing the relatively thick absorbing layer required for photodetection [21,33]. Accordingly, SPLE is used here to prepare PbS-I inks, enabling one-step deposition of compact thick films while reducing process complexity and mitigating LbL-induced stress [21,42].

On top of the PbS-I absorber, a thin PbS-ethane-1,2-dithiol (EDT) layer has been commonly introduced as the p-type/hole-selective cap, as shown in Figure 1a. EDT is commonly used as a short bidentate thiol that strengthens interdot coupling and facilitates hole extraction, and the resulting PbS-I/PbS-EDT bilayer forms an asymmetric junction (often described as p-n or p-i-n-like) that enhances the built-in field and suppresses dark current and interfacial recombination [43]. PbS-EDT is prepared by solid-state ligand exchange (SSLE) on the film, enabling thin interfacial layers and avoiding the colloidal instability of thiol-pre-exchanged inks. Moreover, restricting EDT treatment to a thin cap helps mitigate trap-related losses that can arise in thicker EDT-rich regions [44].

For PbS-I ink preparation, SPLE is first performed to replace native OA with iodide-based ligands, as seen in Figure 1b. First, the fresh PbI_2_ ligand is dissolved in dimethylformamide (DMF) (Step 1) and then combined with an equal volume of OA-capped PbS QD solution in n-octane (Step 2). The mixture is heated in a water bath at temperatures of 25 °C, 40 °C, 50 °C, or 60 °C for 5 min (Step 4), promoting phase separation in which QDs transfer from the nonpolar top layer to the polar bottom layer (Step 5). As seen in Step 5, the ligand solution retains its clear yellow color, similar to the fresh solution, without undergoing additional purification. Notably, heating at 40 °C induces rapid phase separation and ligand exchange within seconds, effectively displacing OA ligands from the QD surface. QD pellets are collected by centrifugation at 5000 rpm for 5 min (Step 6). The resulting solid QDs are redispersed in a butylamine (BTA)/DMF mixture for film deposition. Experimentally, we observe that post-exchange dispersion quality varies due to the adsorption–desorption equilibrium of surface ligands in the solvent environment, and poor colloidal stability often leads to inferior film morphology (e.g., non-uniform coverage or aggregation), which in turn degrades device performance. Thus, ligand exchange is a critical lever for optimizing ink stability, film quality, and device characteristics. Exchange efficiency can be improved by increasing ligand-precursor concentration and/or repeating the exchange steps, though it is ultimately limited by temperature-dependent ligand adsorption isotherms. Given variations in QD surface chemistry arising from synthesis history, identifying an appropriate reaction temperature to control the adsorption isotherm of surface ligands is essential for reproducibly preparing high-quality QD films in devices.

### 3.2. Investigation of Reaction Temperature in OA-Iodide Ligand Exchange

**Figure 2:**
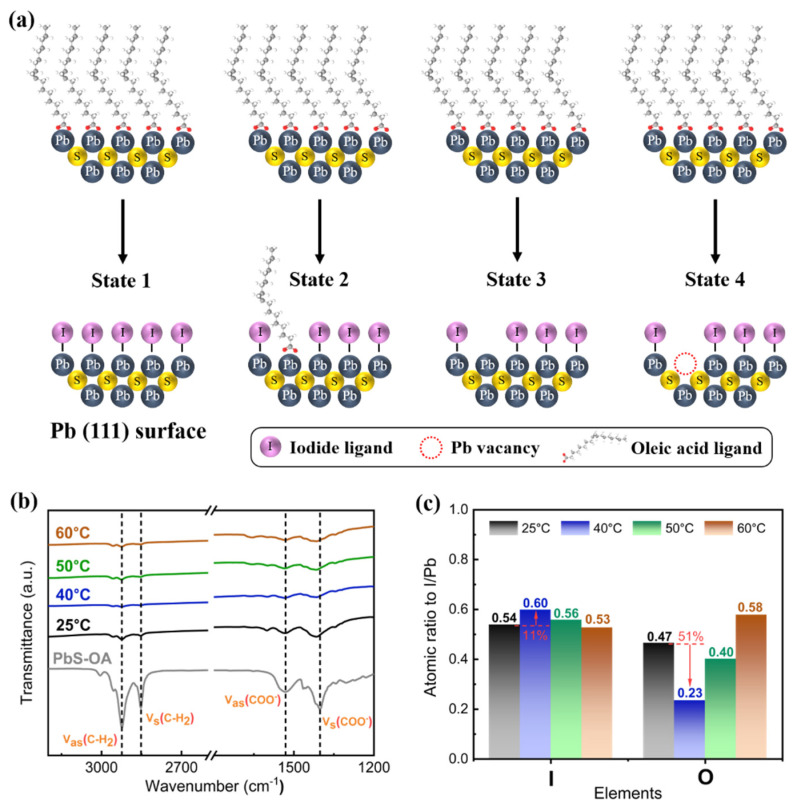
Investigation of OA-iodide ligand exchange temperature. (**a**) Schematic illustration of four possible surface states on Pb-terminated (111)-facet-related surfaces during SPLE. Surface chemistry and binding evolution of PbS QDs after ligand exchange at different temperatures. (**b**) FTIR spectra of PbS-I QD films, with pristine PbS-OA QDs shown for comparison. (**c**) XPS-derived surface composition of PbS-I QD films prepared at different ligand-exchange temperatures.

The SPLE for PbS-I ink generally involves two main steps: initial detachment of OA from QD surfaces, followed by iodide binding (via PbI_2_ treatment) to under-coordinated surface sites, where incomplete exchange or surface atom detachment can generate defect states [21,31]. The focus is on Pb-terminated (111)-facet-related surface chemistry, where SPLE can produce various surface states [17,26,31,35]. As shown in Figure 2a, State 1 illustrates the ideal full exchange scenario, wherein the QD surface is entirely substituted by iodide-derived ligands, which is challenging to achieve experimentally. State 2 retains residual OA post-exchange. State 3 features unpassivated dangling bonds on the QD surface due to incomplete Pb bonding. State 4 involves OA and Pb atom detachment, creating trap states. Reaction temperature governs these outcomes by modulating ligand adsorption–desorption equilibrium and exchange kinetics, profoundly affecting the surface properties of post-exchange PbS-I QD films.

In this work, reaction temperature is examined as a key control parameter for halide incorporation and suppression of oxygen-related species. Figure 2b shows FTIR spectra, where the characteristic vibrational features of native oleate ligands, including the -CH_2_ stretching modes (ν_as_ and ν_s_) and the carboxylate bands (ν_as_ (COO^−^) and ν_s_ (COO^−^)), are attenuated after ligand exchange. This attenuation suggests removal or replacement of OA across the tested temperatures. Figure 2c summarizes the surface composition derived from X-ray photoelectron spectroscopy (XPS). At 40 °C, the ligand-exchange process reaches the most favorable balance between OA removal and iodide incorporation, yielding the most effectively passivated PbS-I surfaces with maximized iodide incorporation and minimized oxygen-related species. The I/Pb ratio increases from 0.54 at 25 °C to 0.60 at 40 °C (~11%), while the O/Pb ratio decreases from 0.47 to 0.23 (~51%). At higher temperatures (50–60 °C), the XPS-derived I/Pb decreases while O/Pb increases, indicating that excessive heating suppresses halide passivation and promotes oxygen-related species. Thus, ligand exchange at 40 °C is selected for reproducible PbS-I ink preparation, enabling well-passivated surfaces and high-quality films for devices.

### 3.3. Electronic Band Structure of Ligand-Exchanged PbS QD Film

**Figure 3:**
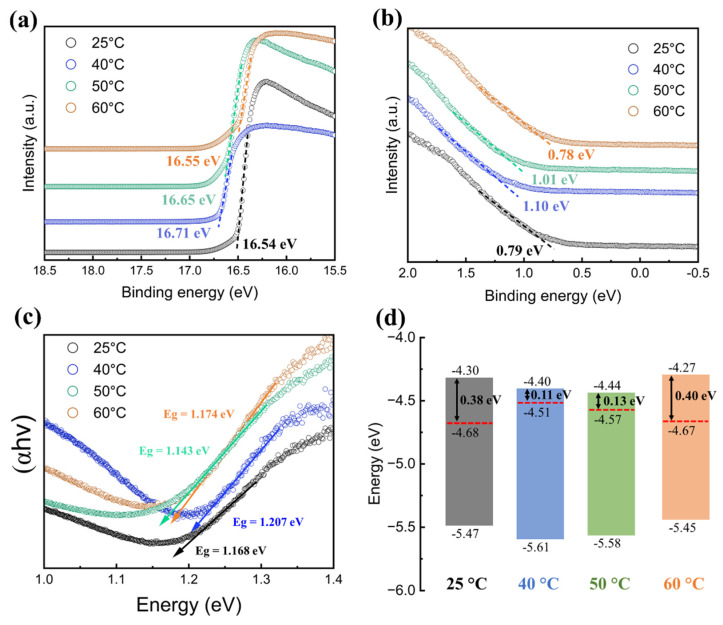
Electronic band structure of SPLE PbS-I QD films prepared at different reaction temperatures (25–60 °C). (**a**) UPS spectra. (**b**) Secondary-electron cut-off region used to determine the work function (ϕ) and Fermi-level position. (**c**) Tauc plots derived from optical absorption spectra to extract the optical bandgap (E_g_). (**d**) Band diagrams constructed from UPS- and Tauc-derived parameters, showing the temperature-dependent alignment of E_F_ relative to the band edges. All energies are referenced to the vacuum level (0 eV).

Ultraviolet photoelectron spectroscopy (UPS) spectra reveal that ligand-exchange temperature tunes the electronic structure of PbS-I films, as seen in Figure 3a,b. The secondary-electron cutoffs shift with temperature, yielding work functions (ϕ) of −4.68 eV (25 °C), −4.51 eV (40 °C), −4.57 eV (50 °C), and −4.67 eV (60 °C) relative to vacuum. These results indicate that the exchange condition modulates both the work function and electronic behavior of the film. In parallel, optical bandgaps derived from Tauc plots in Figure 3c show modest variations (E_g_ = 1.168, 1.207, 1.143, and 1.174 eV for 25, 40, 50, and 60 °C, respectively). The non-monotonic variation in E_g_ across temperatures is attributed to changes in surface passivation quality and interdot electronic coupling rather than QD size variation since all samples are from the same QD batch. Enhanced iodide passivation at 40 °C reduces band-tail states, yielding a slightly larger apparent bandgap, while reduced passivation at higher temperatures partially restores sub-gap absorption. The estimated band diagram is shown in Figure 3d. The offsets from conduction band minimum (CBM, E_C_) to Fermi level (E_F_) are estimated to be around 0.38 eV (25 °C), 0.11 eV (40 °C), 0.13 eV (50 °C), and 0.40 eV (60 °C). These results demonstrate that films exchanged at 40–50 °C exhibit the strongest n-type character (i.e., E_F_ closest to the CBM), peaking at 40 °C. This energetic trend is consistent with the surface-composition optimization (highest I/Pb and lowest O/Pb at 40 °C), supporting 40 °C as the optimal temperature for reproducibly preparing PbS-I films with favorable electron-transport energetics.

### 3.4. Trap-State Density in PbS-I QD Films

Space-charge-limited-current (SCLC) measurements using an electron-dominant (electron-injection-favored) vertical device structure (ITO/PbS-I/Al) are performed to investigate the trap-state density in PbS-I QD films. Based on the energy-level alignment in Figure 3d, the PbS-I films processed at 40–50 °C exhibit a more pronounced n-type character, with the Fermi level much closer to the conduction band (ΔE ≈ 0.11–0.13 eV) than those processed at 25 °C and 60 °C (ΔE ≈ 0.38–0.40 eV). This alignment favors electron injection and supports treating the ITO/PbS-I/Al stack as an electron-dominant structure for V_TFL_-based comparison of relative electron-trap densities. Although this stack is not a perfectly selective electron-only configuration, V_TFL_ is used here as a comparative metric to evaluate relative electron-trap densities across ligand-exchange temperatures under identical device geometry and measurement conditions.

**Figure 4:**
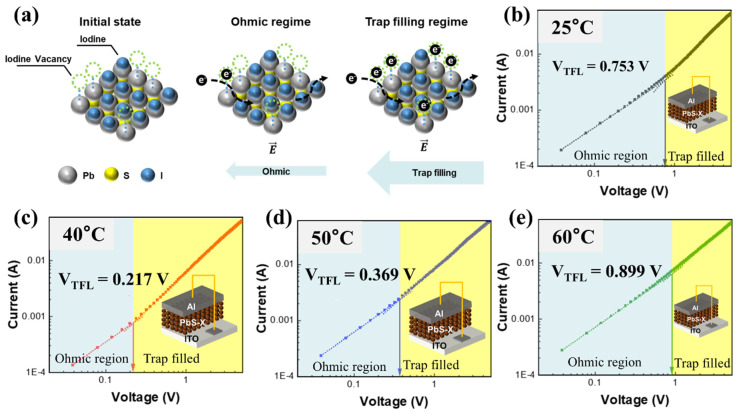
Investigation of trap-state density in SPLE PbS-I QD films using SCLC measurements of an electron-dominant device. (**a**) Schematic illustration of electron transport regimes in SCLC (ohmic, trap-filling, and trap-filled limit). (**b**–**e**) SCLC J-V characteristics of PbS-I QD films prepared with ligand exchange at (**b**) 25 °C, (**c**) 40 °C, (**d**) 50 °C, and (**e**) 60 °C. Inset shows the electron-dominant device architecture ITO/PbS-I/Al.

As illustrated in Figure 4a, iodide-related surface imperfections/defects can serve as electron-trapping sites under an applied electric field. The current-voltage (J-V) characteristics show three typical regimes: an ohmic regime at low bias, a trap-filling regime where injected electrons progressively occupy trap states, and a TFL regime once the traps are saturated [16]. As extracted from Figure 4b–e, the V_TFL_ decreases from 0.753 V (25 °C) to a minimum of 0.217 V (40 °C), and then increases to 0.369 V (50 °C) and 0.899 V (60 °C), highlighting the dependence on ligand-exchange temperature. Because a lower V_TFL_ corresponds to a lower trap density, these results indicate that the 40 °C condition yields the lowest trap density, whereas the 25 °C and 60 °C treatments produce substantially higher trap densities. Notably, this trap-minimization trend is consistent with the surface-chemistry optimization at 40 °C, where halide incorporation is maximized and oxygen-related species are minimized. The extracted V_TFL_ values and the corresponding trap-state densities calculated using the standard TFL relation are summarized in Table 1. Together with the more pronounced n-type character inferred from the band diagrams, these results support 40 °C as the optimal ligand-exchange temperature for producing electronically cleaner PbS-I QD solids.

### 3.5. Photodetection Properties of PbS QDs PD

**Figure 5:**
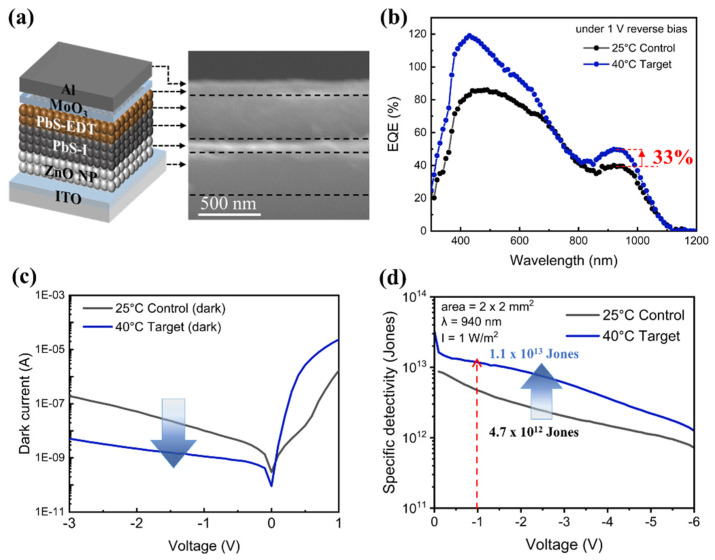
PbS QD PD device. (**a**) Schematic diagram of the device structure. Right inset shows a cross-sectional SEM image of the PD device. (**b**) Variation in EQE values under a bias of −1 V. (**c**) Dark current versus voltage. (**d**) Specific detectivity vs. voltage.

The device adopts an ITO/ZnO/PbS-I/PbS-EDT/MoO_3_/Al architecture, as shown in Figure 5a. The layer thicknesses of ZnO, PbS-I/PbS-EDT, and MoO_3_/Al are estimated ~103 nm, ~353 nm, and ~132 nm. ITO-coated glass serves as the transparent bottom cathode through which NIR light is incident. The ZnO nanoparticle film acts as the electron-transport layer (ETL), selectively extracting electrons from the absorber while blocking holes. The PbS-I layer (acts as n-type) is the primary NIR-absorbing and electron-transporting layer, where iodide passivation reduces surface traps and improves interdot charge transport. A thin PbS-EDT cap (acts as p-type) on top forms an asymmetric n-p heterojunction with PbS-I, promoting built-in-field-driven carrier separation and suppressing dark current. The device is completed by thermally evaporated MoO_3_ as a hole-transport interlayer and Al as the top anode. Compared with the control PbS-I QDs prepared at 25 °C, the device fabricated with QDs ligand-exchanged at 40 °C exhibits improved external quantum efficiency (EQE), as shown in Figure 5b. The EQE is enhanced by ~33% at 940 nm (~52% vs. ~39% at −1 V). Based on the relation of Equation (2)

(2) $$R\left(\lambda \right)=\frac{EQE\cdot q\lambda }{hc}=\frac{EQE\cdot \lambda (nm)}{1240}$$

where R is responsivity, q is the elementary charge, λ is the absorption wavelength, h is Planck’s constant, and c is the speed of light [23,25]. The responsivity at 940 nm is calculated to be ~0.394 A/W for the 40 °C device and ~0.296 A/W for the 25 °C control sample. In addition, the 40 °C device shows a reduced dark current ~1.05 × 10^−9^ A/cm^2^ at −1 V (Figure 5c), compared with the control (~1 ×10^−8^ A/cm^2^). Detectivity is estimated under the shot-noise-limited assumption using Equation (3),

(3) $$D^{*}\approx R/\sqrt{2qJ_{d}}$$

where 2qJ_d_ denotes the shot-noise current spectral density per unit bandwidth under the shot-noise-limited approximation. Because the measured noise current can also include contributions from thermal and flicker noise, the detectivity values reported here are estimated as specific detectivities using this approach. Although direct measurements of the noise spectral density would provide a more rigorous evaluation for practical device operation, this method is widely used in QD PD studies to assess material-level improvements and to provide a reasonable relative indication of dark current suppression [23,25,29]. The detectivity at 940 nm of the target device at 40 °C is estimated to be ~2.1 × 10^13^ Jones at −1 V, compared with ~4.7 × 10^12^ Jones for the 25 °C control, corresponding to an approximately 4.5-fold improvement. The results illustrate that improved halide passivation mitigates defect-mediated carrier trapping, recombination and leakage pathways. This is consistent with the concurrent EQE/responsivity enhancement and dark-current suppression under the temperature-optimized ligand-exchange condition.

## 4. Conclusions

This study establishes reaction-temperature control as a decisive lever for liquid-phase OA-to-halide ligand exchange in PbS QDs and demonstrates how optimized surface chemistry translates into improved electronic quality and photodetection performance. Among the tested conditions, 40 °C yields the most favorable surface composition by maximizing halide incorporation and minimizing oxygen-related species, which is consistent with enhanced n-type energetics and the lowest trap signature in SCLC (V_TFL_ = 0.217 V). This optimized ligand-exchange condition simultaneously increases charge collection and suppresses leakage, delivering 52% EQE at 940 nm under −1 V (vs. 39% for the 25 °C control), a corresponding responsivity of 0.394 A/W, and a markedly reduced dark current density of 1.05 × 10^−9^ A/cm^2^. The detectivity is estimated (shot-noise-limited) to be ~2.1 × 10^13^ Jones at 940 nm and −1 V, 4.5-fold higher than the control. Overall, temperature-optimized liquid-phase ligand exchange provides a practical and reproducible route to halide-passivated PbS inks and high-performance PbS QD PDs.

## Figures and Tables

**Table 1:** Summary of trap-filled-limit voltages V_TFL_ extracted from SCLC measurements and the corresponding trap-state densities of PbS-I QD films (thickness ∼200 nm). Effective relative permittivity is treated as a constant across samples.

	25 °C	40 °C	50 °C	60 °C
V_TFL_ (V)	0.753	0.217	0.369	0.899
Trap density (cm^−3^)	3.75 × 10^16^	1.08 × 10^16^	1.84 × 10^16^	4.47 × 10^16^

## Data Availability

The original contributions presented in this study are included in the article. Further inquiries can be directed to the corresponding author.
